# IL-1β promotes esophageal squamous cell carcinoma growth and metastasis through FOXO3A by activating the PI3K/AKT pathway

**DOI:** 10.1038/s41420-024-02008-0

**Published:** 2024-05-18

**Authors:** Shuangshuang Chen, Ying Yang, Zhaoyang Zheng, Man Zhang, Xixian Chen, Nan Xiao, Hongchun Liu

**Affiliations:** 1 The Second Clinical Medical College of Henan University of Chinese Medicine, Zhengzhou, Henan 450002 China; 2 Department of Clinical Laboratory, First Affiliated Hospital of Zhengzhou University, Zhengzhou, Henan 450052 China

**Keywords:** Oesophageal cancer, Mechanisms of disease

## Abstract

Esophageal cancer is a common type of cancer that poses a significant threat to human health. While the pro-inflammatory cytokine IL-1β has been known to contribute to the development of various types of tumors, its role in regulating esophageal cancer progression has not been extensively studied. Our studies found that the expression of IL-1β and FOXO3A was increased in esophageal squamous cell carcinoma (ESCC). IL-1β not only increased the proliferation, migration, and invasion of two ESCC cell lines but also promoted tumor growth and metastasis in nude mice. We also observed that IL-1β and FOXO3A regulated the process of epithelial-mesenchymal transition (EMT) and autophagy. The PI3K/AKT pathway was found to be involved in the changes of FOXO3A with the expression level of IL-1β. The AKT agonist (SC79) reversed the reduction of FOXO3A expression caused by the knockdown of IL-1β, indicating that IL-1β plays a role through the PI3K/AKT/FOXO3A pathway. Furthermore, the knockdown of FOXO3A inhibited ESCC development and attenuated the pro-cancer effect of overexpressed IL-1β. Targeting IL-1β and FOXO3A may be potentially valuable for the diagnosis and treatment of ESCC.

## Introduction

Esophageal cancer is a malignant tumor of the digestive system that is becoming more prevalent among people worldwide, ranking seventh in the world in terms of incidence and sixth in terms of mortality [1], with a 5-year survival rate as low as 19% [2]. There are two major histological subtypes of esophageal cancer: esophageal adenocarcinoma and ESCC [3]. While the incidence of esophageal adenocarcinoma and its precursor lesion, Barrett’s esophagus, has increased in Western populations over the past four decades, ESCC continues to account for the vast majority of all esophageal cancer cases diagnosed annually worldwide [4]. Patients diagnosed with ESCC at an early stage and treated with active and rational anticancer therapy have a good chance of achieving a clinical cure. However, most patients are already in intermediate or advanced stages at the time of diagnosis, making surgical treatment less effective [5–7]. Despite the use of various therapeutic approaches, such as surgical resection, chemotherapy, and radiotherapy, the prognosis of ESCC patients remains poor [8–10]. Therefore, elucidating the underlying molecular mechanisms leading to the development of ESCC is crucial for the development of more innovative and effective therapeutic approaches.

IL-1β is a typical pro-inflammatory cytokine, which both exerts innate immunity against invasion of pathogenic microorganisms and promotes autoimmune diseases and tumorigenesis [11–14]. IL-1β produced during chronic inflammatory processes has been reported to support tumor development [15–17]. In addition, IL-1β infiltrated in the tumor microenvironment promotes tumor growth and metastasis by promoting the expression of IL-1 targets associated with neoangiogenesis as well as soluble mediators in cancer-associated fibroblasts that cause anti-apoptotic signaling [18]. The pro-cancer effects of IL-1β have been widely demonstrated, but few studies have been reported on the oncogenic mechanisms of IL-1β in esophageal cancer.

Our study revealed the expression and function of IL-1β in ESCC, and that IL-1β may regulate ESCC cell proliferation, migration, and invasion by activating the PI3K/AKT/FOXO3A signaling pathway, promoting EMT and inhibiting autophagy.

## Results

### IL-1β was upregulated in ESCC and associated with poor prognosis

**Fig. 1:**
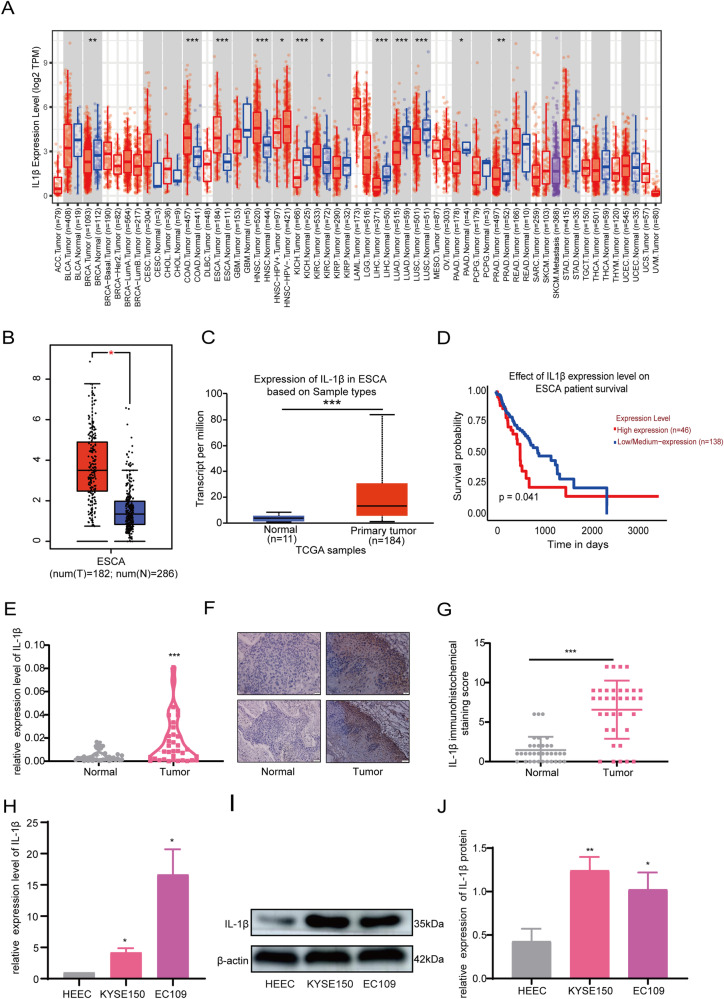
IL-1β was upregulated in ESCC. **A**–**C** The TIMER, GEPIA, and UALCAN databases were used to analyze IL-1β expression in esophageal cancer. **D** The UALCAN database was used for survival analysis to reveal the relationship between IL-1β expression and prognosis of esophageal cancer patients. **E**–**G** The expression of IL-1β in ESCC tumor tissues and paracancerous tissues was examined by RT-qPCR and IHC. **H**–**J** The expression of IL-1β in HEEC, KYSE150, and EC109 was detected by RT-qPCR and western blot. Error bars stand for mean ± standard deviation (SD). *P < 0.05, **P < 0.01, ***P < 0.001.

In this study, the expression of IL-1β in esophageal cancer was first preliminarily analyzed using the TIMER, GEPIA, and UALCAN databases. As shown, the expression of IL-1β in esophageal cancer was significantly higher than that in normal tissues (*P* < 0.05, Fig. 1A–C). Survival analysis of IL-1β by the UALCAN database indicated that the survival rate of patients with high expression of IL-1β was significantly lower than that of patients with low expression (*P* < 0.05, Fig. 1D). To investigate the expression of IL-1β in ESCC, RT-qPCR, and IHC were performed to detect the mRNA and protein levels of IL-1β in clinical specimens from 35 ESCC patients who had undergone surgical resection between September 2021 and December 2021 at the First Affiliated Hospital of Zhengzhou University. The results found that IL-1β expression was significantly elevated in ESCC cancer tissues compared with paracancerous tissues (*P* < 0.001, Fig. 1E–G). Patients with high IL-1β expression may have larger tumor size, poorer differentiation, and a higher number of lymph node metastases compared with the low expression group (*P* < 0.05, Table 1). In addition, we examined the expression of IL-1β in normal esophageal epithelial cells HEEC and ESCC cells by RT-qPCR and Western blot. The findings demonstrated that IL-1β expression levels were higher in KYSE150 and EC109 compared with HEEC (*P* < 0.05, Fig. 1H–J).

**Table 1:** Relationship between IL-1β expression and tumor clinicopathological features in ESCC patients.

Features	IL-1β expression		*P* value
	High (23)	Low (12)	
Gender			
Male (23)	17	6	0.261
Female (12)	6	6	
Age			
≥60 (29)	18	11	0.640
<60 (6)	5	1	
Tumor size			
≥3 cm (24)	20	4	0.002**
<3 cm (11)	3	8	
Differentiation grade			
G1 (11)	4	7	0.022*
G2/G3 (24)	19	5	
Lymphatic metastasis			
N0 (11)	3	8	0.002**
N1–N2 (24)	20	4	

*ESCC* esophageal squamous cell carcinoma.

*p < 0.05, **p＜0.01.

### IL-1β promoted the development of ESCC in vitro

**Fig. 2:**
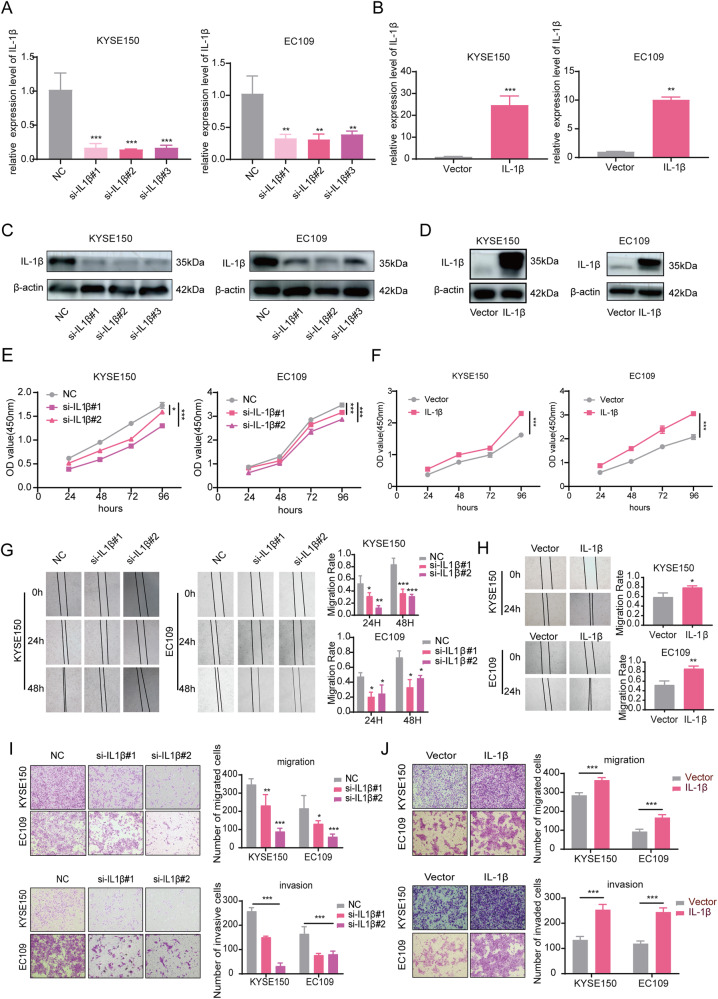
IL-1β promoted the development of ESCC. **A**–**D** The knockdown and overexpression efficacy of IL-1β in KYSE150 and EC109 was verified by RT-qPCR and Western blot. **E**, **F** The CCK-8 assay was performed to examine the effects of knockdown and overexpression of IL-1β on ESCC cell proliferation. **G**–**J** The effects of knockdown and overexpression of IL-1β on the migration and invasion ability of ESCC cells were assessed by the scratch assay and the Transwell assay. All data are expressed as the mean ± SD of values from experiments performed in triplicate. *P < 0.05, **P < 0.01, ***P < 0.001.

To understand the role of IL-1β in tumor progression, we transfected KYSE150 and EC109 with siRNA and lentivirus to construct cellular models for knockdown and overexpression of IL-1β. RT-qPCR showed significant knockdown and overexpression efficacy of IL-1β in both KYSE150 and EC109 (*P* < 0.01, Fig. 2A, B), which was further confirmed by Western blot (Fig. 2C, D). The CCK-8 assay and the colony formation assay revealed that knockdown of IL-1β inhibited the proliferation of KYSE150 and EC109, while overexpression of IL-1β promoted the proliferation (*P* < 0.05, Fig. 2E, F, Fig. S1A, B). Moreover, knockdown or overexpression of IL-1β significantly inhibited or enhanced the migration and invasion of KYSE150 and EC109 (*P* < 0.05, Fig. 2G–J). Therefore, our findings suggest that IL-1β plays a crucial role in promoting the development of ESCC in vitro.

### IL-1β promoted poor progression of ESCC through activation of the PI3K/ AKT/FOXO3A pathway

**Fig. 3:**
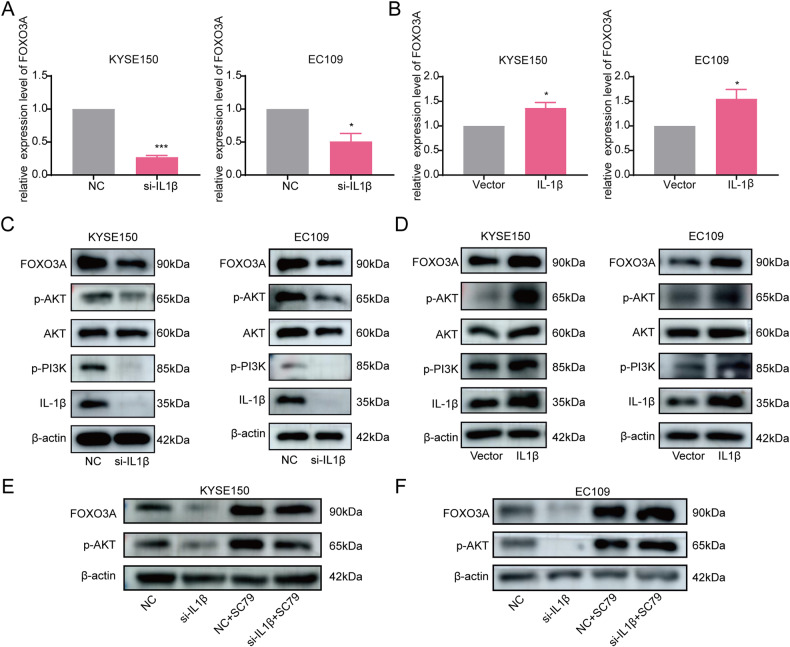
IL-1β regulated the PI3K/AKT/FOXO3A signaling pathway. **A**, **B** The impact of IL-1β knockdown and overexpression on FOXO3A mRNA expression was detected by RT-qPCR. **C**, **D** Western blot was used to detect the effect of IL-1β knockdown and overexpression on the expression of p-PI3K, AKT, p-AKT, and FOXO3A. **E**, **F** The effect of AKT agonist SC79 on FOXO3A expression was examined by Western blot. All data are expressed as the mean ± SD of values from experiments performed in triplicate. *P < 0.05, ***P < 0.001.

We observed that the mRNA expression of FOXO3A was decreased upon IL-1β knockdown and increased upon IL-1β overexpression (*P* < 0.05, Fig. 3A, B). Western blot also observed a decrease in FOXO3A protein expression after IL-1β knockdown (Fig. S2). To understand how IL-1β-dependent FOXO3A expression occurs in ESCC cells, we examined the impact of IL-1β on the phosphorylation of PI3K and AKT. The data demonstrated that the expression of p-PI3K and p-AKT were significantly reduced in the si-IL-1β group compared with the NC group, and the opposite result was obtained by overexpressing IL-1β (Fig. 3C, D). In addition, the addition of the AKT agonist SC79 to the si-IL-1β group reversed the reduction of p-AKT and FOXO3A caused by IL-1β knockdown (Fig. 3E, F). Thus, we concluded that IL-1β hurts ESCC through the PI3K/AKT/FOXO3A pathway.

### IL-1β was involved in EMT and autophagy processes

**Fig. 4:**
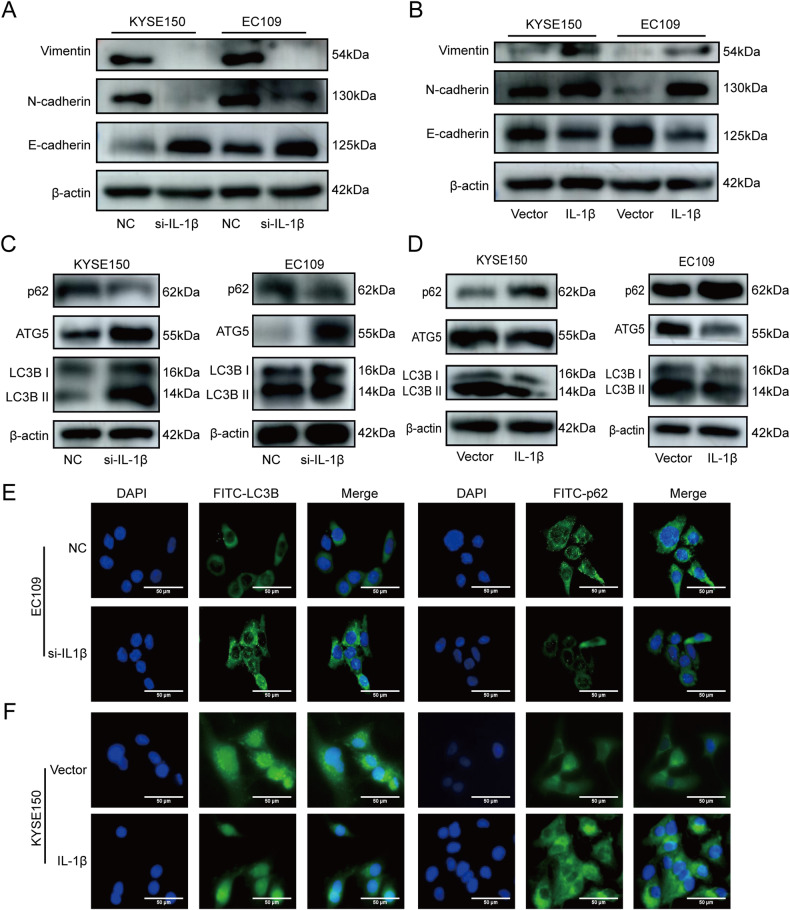
IL-1β promoted EMT and inhibited autophagy. **A**, **B** Western blot was performed to detect the expression of E-cadherin, N-cadherin, and Vimentin in KYSE150 and EC109 with knockdown and overexpression of IL-1β. **C**, **D** LC3BII/I, ATG5, and P62 in KYSE150 and EC109 with knockdown and overexpression of IL-1β were tested by Western blot. **E**, **F** IF was used to determine the aggregation degree of LC3B and P62 in KYSE150 and EC109 with knockdown and overexpression of IL-1β.

Next, we explored the expression of E-cadherin, N-cadherin, and Vimentin in KYSE150 and EC109 with knockdown and overexpression of IL-1β, which are important EMT markers associated with migration and invasion. Western blot results showed increased expression of E-cadherin and decreased expression of N-cadherin, and Vimentin in the si-IL-1β group compared with the NC group. Overexpression of IL-1β yielded results opposite to the above (Fig. 4A, B). Thus, IL-1β may promote ESCC migration and invasion by regulating EMT. Furthermore, the expression of LC3BII/I, ATG5, beclin1, and P62, which are markers associated with autophagy, was analyzed using Western blot in KYSE150 and EC109 with knockdown and overexpression of IL-1β. The results indicated that, compared with the NC group, the expression of LC3BII/I, ATG5, and beclin1 were increased in the si-IL-1β group, while the expression of P62 was decreased in the si-IL-1β group (Fig. 4C, Fig. S3). Overexpression of IL-1β yielded results opposite to those described above (Fig. 4D). Additionally, IF was used to assess the impact of IL-1β on autophagy in ESCC cells by detecting the aggregation degree of LC3B and P62, and the results were consistent with those obtained from Western blot (Fig. 4E, F). Thus, IL-1β may play a role in ESCC progression by regulating autophagy.

### IL-1β promoted ESCC growth and metastasis in vivo

**Fig. 5:**
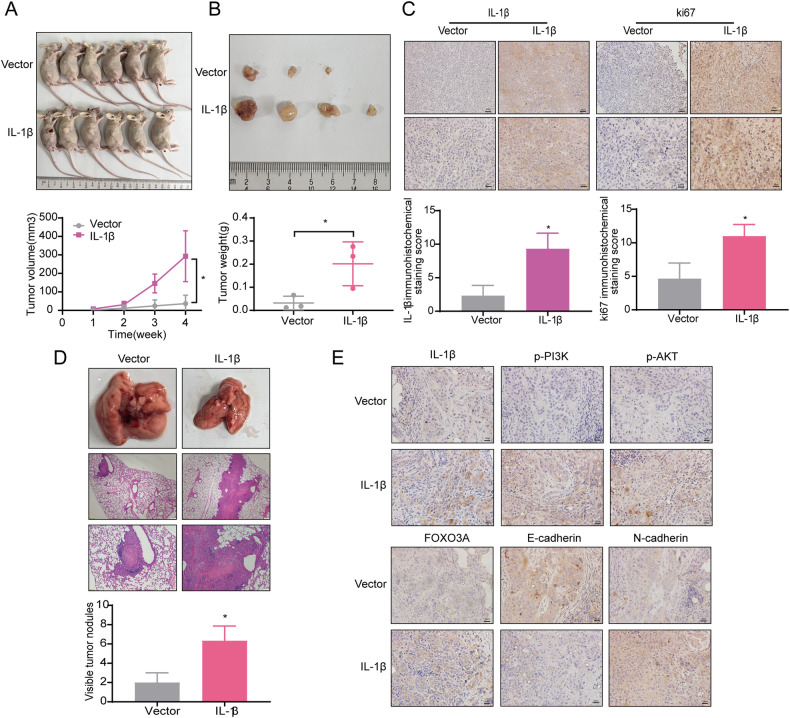
IL-1β promoted tumor growth and metastasis in vivo. **A** KYSE150 overexpressing IL-1β was injected subcutaneously into the right rib of nude mice to assess tumorigenesis. Tumor volumes were measured and recorded weekly. **B** Tumors removed from the executed nude mice were photographed and weighed. **C** IHC was used to analyze IL-1β and ki67 expression in tumors stripped from nude mice. **D** KYSE150 overexpressing IL-1β was injected into the tail vein of nude mice, and the lungs were dissected and weighed 28 days later. Lung metastasis was assessed by HE staining. **E** IHC was conducted to compare the expression of IL-1β, p-PI3K, p-AKT, FOXO3A, N-cadherin, and E-cadherin in lung tissues. Error bars stand for mean ± standard deviation (SD). *P < 0.05.

To confirm the effect of IL-1β on ESCC growth in vivo, we established a subcutaneous tumorigenic model in nude mice by injecting KYSE150 overexpressing IL-1β. Measurement of tumor volume throughout the culture of the animal model manifested that tumor growth was significantly accelerated in the overexpression of the IL-1β group compared with the vector group (*P* < 0.05, Fig. 5A). After the nude mice were euthanised, we measured the weight of the tumors and found that they were heavier in the overexpression of the IL-1β group (*P* < 0.05, Fig. 5B). The IHC results displayed that the expressions of IL-1β and ki67 in the overexpressed IL-1β group were markedly higher than those in the Vector group (Fig. 5C). To further determine the effects of IL-1β on the PI3K/AKT/FOXO3A signaling pathway and metastasis in vivo, we established a lung metastasis model by injecting KYSE150 overexpressing IL-1β into the tail vein of nude mice. The results of HE staining proved that the number of metastatic nodules in the lung tissues of nude mice injected with IL-1β cells was significantly higher than that in the control group (*P* < 0.05, Fig. 5D). IHC exhibited increased expression of IL-1β, p-PI3K, p-AKT, FOXO3A, and N-cadherin and decreased expression of E-cadherin in lung tissues overexpressing IL-1β (Fig. 5E), a result which reinforces the conclusion that IL-1β can promote the development of ESCC through the PI3K/AKT/FOXO3A signaling pathway and EMT.

### Knockdown of FOXO3A inhibited the development of ESCC

**Fig. 6:**
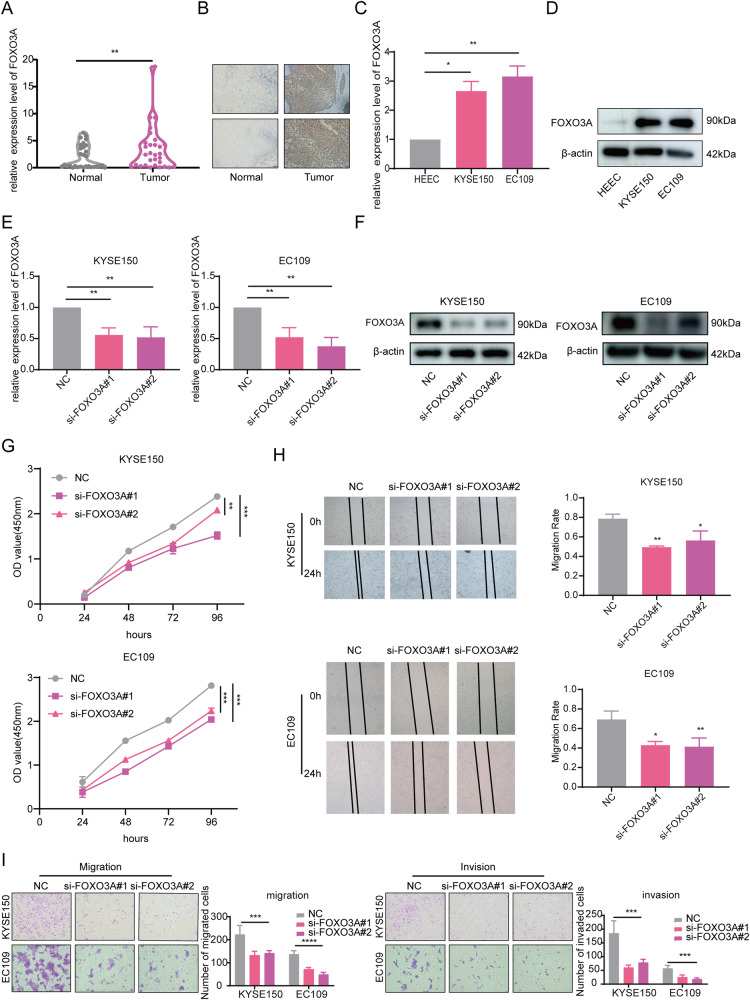
Knockdown of FOXO3A inhibited ESCC development. **A**, **B** The expression of FOXO3A in ESCC tumor tissues and paracancerous tissues was detected by RT-qPCR and IHC. **C**, **D** RT-qPCR and Western blot were used to test the expression of FOXO3A in HEEC, KYSE150, and EC109. **E**, **F** The efficiency of FOXO3A knockdown was verified by RT-qPCR and Western blot. **G** The CCK-8 assay was performed to examine the impact of FOXO3A knockdown on cell proliferation. **H**, **I** The effect of FOXO3A knockdown on the migration and invasion ability of ESCC cells was assessed by the Scratch assay and the Transwell assay. All data are expressed as the mean ± SD of values from experiments performed in triplicate. *P < 0.05, **P < 0.01, ***P < 0.001.

Furthermore, we used IHC and RT-qPCR to detect FOXO3A expression in clinical specimens from 30 ESCC patients. The results showed that tumor tissues had significantly higher FOXO3A expression than paracancerous tissues (*P* < 0.01, Fig. 6A, B). Patients with high expression of FOXO3A were more likely to have worse differentiation and a higher number of lymph node metastases compared with the low expression group (*P* < 0.05, Table 2). Additionally, RT-qPCR and Western blot results showed that FOXO3A expression was higher in KYSE150 and EC109 compared with HEEC. (*P* < 0.05, Fig. 6C, D). Similarly, to investigate the function of FOXO3A in tumor progression, we created a cellular model for the knockdown of FOXO3A by transfecting si-RNA into KYSE150 and EC109. The knockdown efficiency of FOXO3A was confirmed through RT-qPCR (*P* < 0.01, Fig. 6E) and Western blot (Fig. 6F). The CCK-8 assay indicated that the proliferation ability of KYSE150 and EC109 was diminished after the knockdown of FOXO3A (*P* < 0.01, Fig. 6G). The Scratch assay and the Transwell assay were performed to evaluate the effect of FOXO3A knockdown on cell migration and invasion, and the results showed that the cell migration and invasion abilities were significantly reduced in the si-FOXO3A group compared with the NC group (*P* < 0.05, Fig. 6H, I). In conclusion, the knockdown of FOXO3A inhibited the development of ESCC.

**Table 2:** Relationship between FOXO3A expression and tumor pathological features in ESCC patients.

Features	FOXO3A expression		p Value
	High (20)	Low (10)	
Gender			
Male (19)	14	5	0.425
Female (11)	6	5	
Age			
≥60 (25)	17	8	1.000
<60 (5)	3	2	
Tumor size			
≥3 cm (22)	17	5	0.078
<3 cm (8)	3	5	
Differentiation grade			
G1 (9)	3	6	0.030*
G2/G3 (21)	17	4	
Lymphatic metastasis			
N0 (9)	2	7	0.002**
N1-N2 (21)	18	3	

*ESCC* esophageal squamous cell carcinoma.

*p < 0.05; **p < 0.01.

### Knockdown of FOXO3A alleviated the effect of overexpressing IL-1β on ESCC

**Fig. 7:**
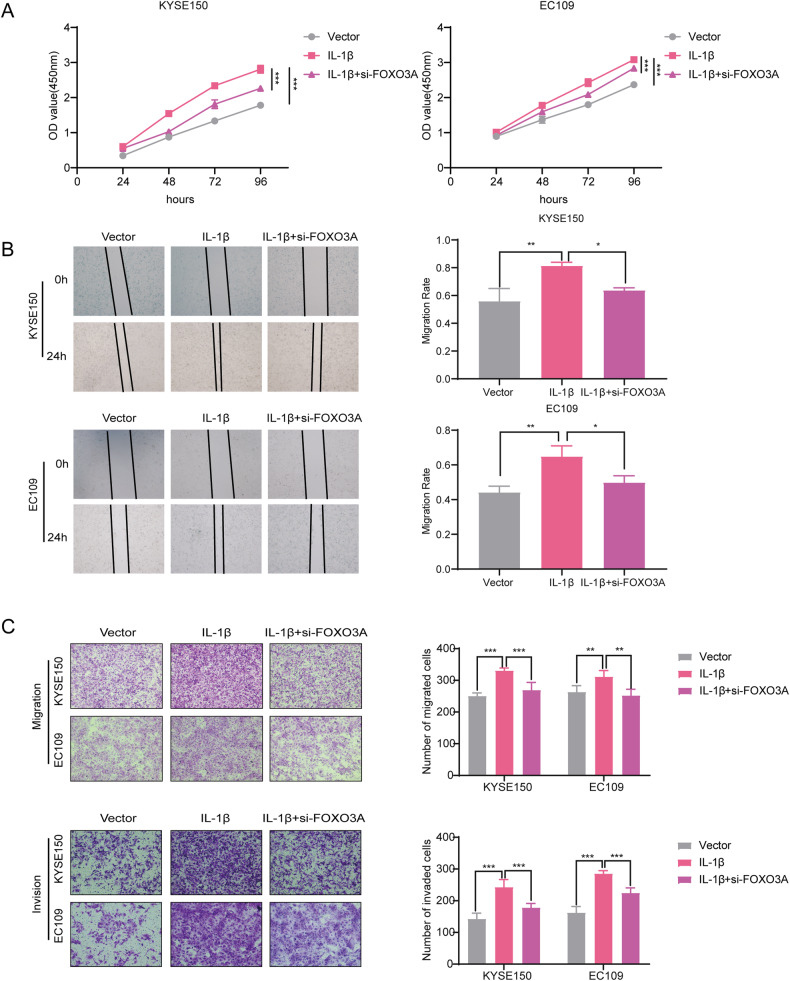
Knockdown of FOXO3A attenuated the effect of overexpressing IL-1β on ESCC. **A** The CCK-8 assay was conducted to examine the effect of knockdown of FOXO3A on the proliferation of ESCC cells overexpressing IL-1β. **B**, **C** The effect of knocking down FOXO3A on the migration and invasion ability of ESCC cells overexpressing IL-1β was assessed by the scratch assay and the Transwell assay. All data are expressed as the mean ± SD of values from experiments performed in triplicate. *P < 0.05, **P < 0.01, ***P < 0.001.

The cell function experiments were performed in ESCC cells overexpressing IL-1β transfected with si-FOXO3A. The results of CCK-8 assay showed that cell proliferation was attenuated in the IL-1β+siFOXO3A group compared with the IL-1β group (*P* < 0.001, Fig. 7A). Similarly, the scratch assay and the Transwell assay results indicated that the cell migration and invasion abilities were also reduced in the IL-1β+siFOXO3A group compared with the IL-1β group (*P* < 0.05, Fig. 7B, C). Therefore, knocking down FOXO3A could alleviate the promoting effect of overexpressing IL-1β on the proliferation, migration, and invasion ability of ESCC cells to some extent.

## Discussion

Esophageal cancer is a prevalent and malignant tumor globally, ranking fourth among all cancers in China [19]. Shockingly, statistics from 2018 showed that 1 in every 20 cancer deaths is due to esophageal cancer [20]. ESCC is responsible for about 90% of all esophageal cancer cases, which is extremely aggressive and fatal, and has a very poor prognosis [21]. Despite extensive research that has been invested in markers affecting the proliferation and metastasis of ESCC [22–24], the morbidity and mortality rates of ESCC have not been effectively improved. Therefore, elucidating the underlying molecular mechanisms that promote the development of ESCC will help to develop more innovative approaches, which are important for establishing effective targeted therapies, increasing the cure rate of ESCC, and reducing the mortality rate.

It has been reported that primary breast cancer patients with increased IL-1β expression are more likely to develop bone metastasis [25, 26]. It has been found that large amounts of IL-1β are present in tumors, which are mainly produced by immune or malignant cells in the tumor microenvironment (TME) and severely influence the course of malignant tumors. Tumor-associated macrophages (TAM) are important immune cells constituting the TME and have both M1 and M2 phenotypes. M1-type macrophages mainly secrete pro-inflammatory cytokines, whereas M2-type macrophages mainly secrete anti-inflammatory cytokines. The latest report suggests that TAM and tumor monocytes are the main sources of IL-1β in human pancreatic ductal adenocarcinoma, which is closely related to the malignant progression of human pancreatic ductal adenocarcinoma and the poor prognosis of patients [27]. IL-1β secreted by M1 macrophages can mediate the immune escape of tumor cells and thus play a pro-carcinogenic role by inducing the expression of the programmed cell death ligand PD-L1 in hepatocellular carcinoma [28]. IL-1β may also act synergistically with IFN-γ to promote maximal upregulation of PD-L1 in non-small cell lung cancer cells through activation of MAPK signaling thereby producing immunosuppressive effects [29]. EMT has been recognized as a key factor in the promotion of tumor metastasis, IL-1β and transforming growth factor β2 (TGF-β2) have been found to decrease epithelial cell markers and increase mesenchymal cell markers in normal human esophageal microvessel endothelial cells, enhancing cell proliferation and migration properties [30]. Additionally, IL-1β in combination with IL-1 receptor antagonist (IL-1RA) regulates EMT by affecting autophagy, which promotes a series of undesirable biological behaviors such as proliferation, migration, and invasion of colorectal cancer cells [31].

Our study found that the expression of IL-1β was significantly higher in ESCC tumor tissues than in paracancerous tissues, which aligns with the results obtained from the TIMER, GEPIA, and UALCAN database. In vitro experiments showed that knockdown of IL-1β significantly inhibited cell proliferation, migration, and invasion, while overexpression of IL-1β exhibited the opposite effect, indicating the role of IL-1β as a tumor promoter in ESCC. The results of animal experiments showed that overexpression of IL-1β led to the promotion of tumor growth and metastasis in vivo, suggesting that IL-1β could be a potential therapeutic target for ESCC treatment. EMT is a biological process of phenotypic transition from epithelial to mesenchymal cells, which usually causes a decrease in intercellular adhesion and an increase in cell migratory motility, and is closely related to tumor invasion, metastasis, and treatment resistance [32]. We discovered that knockdown of IL-1β upregulated the expression of E-cadherin, and down-regulated the expression of N-cadherin and vimentin, overexpression of IL-1β yielded the opposite results. Autophagy and autophagy-associated (ATG) proteins play an important role in cancer development, both by preventing tumourigenesis and inhibiting progression in the early stages of cancer, and by promoting tumor growth, metastasis, and invasion in the late stages of cancer [33]. LC3B-II and ATG5 have key roles in the formation of autophagosomes and autophagic vesicles. ATG5 is involved in the phases of autophagosome initiation, nucleation, extension, and closure [34, 35], whereas LC3B-II is the only protein retained in the bilayer membrane of autophagosomes during autophagosome ontogeny and development, and it is the most direct evidence for the confirmation of cellular autophagy [36]. In addition, p62 is a widely studied substrate for autophagy. During autophagosome formation, p62 acts as a bridge between LC3B and polyubiquitinated proteins and is selectively wrapped into the autophagosome, after which it is degraded by proteolytic hydrolases in the autophagic lysosome [37]. Therefore, the expression of the p62 protein is negatively correlated with autophagic flux. Our study found that LC3BII/I ratio and ATG5 expression were increased and P62 expression was decreased in the knockdown group compared with the control group; overexpression of IL-1β obtained the opposite results to those mentioned above. In addition, the effect of IL-1β on autophagy in ESCC cells was assessed by immunofluorescence assay to detect the aggregation degree of LC3B and p62, and it was found that the aggregation degree of LC3B was enhanced and that of p62 was weakened in EC109 cells knocked down with IL-1β, and that LC3B aggregation degree was weakened and p62 aggregation degree was enhanced in KYSE150 cells overexpressing IL-1β. Therefore, IL-1β may play a role in ESCC progression by regulating autophagy.

The PI3K/AKT signaling pathway is frequently activated in a variety of tumors and plays a crucial role in tumor cell proliferation, apoptosis, metastasis, and other malignant behaviors [38–42]. It has been found that IL-1β facilitates the expression of vasculogenic mimicry markers in breast cancer cells by activating the PI3K / Akt signaling pathway, exerting adverse effects on tumor angiogenesis and cancer cell invasion and metastasis [43]. FOXO3A is a downstream molecule of the PI3K/AKT signaling pathway, which is associated with the development, progression, and prognosis of various tumors. When the PI3K/AKT signaling pathway is activated, p-AKT phosphorylates three phosphorylation sites of FOXO3A, leading to the binding of the phosphorylated FOXO3A transcription factor to the 14-3-3 protein. This complex moves FOXO3A from the nucleus to the cytoplasm of the cell, inhibiting the transcriptional activity of FOXO3A, and promoting cell proliferation and differentiation [44]. While the roles and mechanisms of FOXO3A in the development and progression of breast cancer [45], colon cancer [46], pancreatic cancer [47], lung cancer [48], and oral squamous cell carcinoma [49] have been reported in detail, little is known about its molecular mechanisms in mediating the development and prognosis of ESCC.

Our study found that FOXO3A expression is influenced by IL-1β levels. Knockdown of IL-1β resulted in decreased FOXO3A expression while overexpression of IL-1β increased FOXO3A overexpression. Moreover, treatment with the AKT agonist SC79 was able to reverse the reduction in FOXO3A expression levels caused by IL-1β knockdown. Therefore, we concluded that FOXO3A is a potential downstream target of IL-1β. Further validation showed that FOXO3A expression was upregulated in ESCC tumor tissues compared to paracancerous tissues. In vitro cellular experiments also demonstrated that knockdown of FOXO3A inhibited ESCC cells proliferation, migration, and invasion. More importantly, we further illustrated that knockdown of FOXO3A attenuated the promotion of IL-1β overexpression in ESCC, again suggesting its role as a downstream target of IL-1β.

## Conclusion

In conclusion, the present data suggested that IL-1β and FOXO3A were upregulated in ESCC and were significantly linked to worse prognosis. IL-1β promoted ESCC development, which can be achieved by activating the PI3K/AKT/FOXO3A signaling pathway, promoting EMT processogenesis and inhibiting autophagy.

## Materials and methods

### Tissue specimens and cell lines

Between September 2021 and December 2021, paired cancer tissue and paracarcinoma tissue specimens from 35 ESCC patients who did not receive preoperative radiotherapy or chemotherapy were collected from the First Affiliated Hospital of Zhengzhou University. The study involving clinical samples was reviewed and approved by the Ethics Committee of the First Affiliated Hospital of Zhengzhou University (2021-KY-1131-002). Patients signed an informed consent form. The human normal esophageal epithelial cells (HEEC) and ESCC cell lines (KYSE150 and EC109) were purchased from the Cell Bank of the Chinese Academy of Sciences (Shanghai, China). All cell lines used in the study were identified by STR and excluded from mycoplasma contamination. All cells were cultured in 1640 medium (Gibco, NY, USA) supplemented with 10% fetal bovine serum (Gibco, NY, USA) at 37 °C in a 5% CO_2_ incubator.

### Reagents

β-actin antibody (GB11113-100) and IL-1β (GB15003-100) antibody were purchased from Servicebio, LC3B antibody (T55992), ATG5 antibody (T55766), AKT antibody (T55561) and p-AKT antibody (T40067) were purchased from Abmart. p-PI3K antibody (CY6427) and ki67 antibody (CY5542) were purchased from Abways. FOXO3A antibody (10849-1-AP), p62 antibody (18420-1-AP), E-cadherin antibody (20874-1-AP), N-cadherin antibody (22018-1-AP), Vimentin antibody (10366-1-AP) and HRP -conjugated Affinipure Goat Anti-Rabbit IgG (SA00001-2) were purchased from proteintech. Lipofectamine 3000 (L3000015) was purchased from Thermo Fisher Scientific. AKT agonist SC79 (SF2703-10mM) was purchased from Beyotime. Puromycin solution (E607054-0001) was purchased from sangon.

### Immunohistochemistry (IHC)

The tissue sections were first baked in a 70° oven for 30 min and then treated with xylene to remove paraffin. They were then hydrated with various ethanol concentrations and antigenically repaired with citrate buffer and 3% H_2_O_2_ to remove the effect of endogenous peroxidase. After 10 min of goat serum occlusion, primary antibodies were added dropwise to all sections and left to incubate at 4 °C overnight. Then, horseradish peroxidase (HRP)-labeled secondary antibody was added and incubated for 1 h at room temperature. The DAB working solution was prepared according to the instructions in the DAB kit to render the color of the sections. The IHC staining results were analyzed using the product of the positive cell percentage score and the staining intensity score.

### RNA extraction and reverse‐transcription quantitative PCR (RT-qPCR) reaction

The TRIZol Total RNA Extraction Reagent (Ambion, Texas, USA) was used to extract total RNA from tissues and cells. Reverse transcription (Kemix, Zhengzhou, China) and quantitative amplification (Kemix, Zhengzhou, China) were respectively performed following the manufacturer’s instructions. β-actin was used as an internal reference, and the relevant primers used for RT-qPCR reactions were designed and synthesized by Sangon Biotech (Shanghai, China). The primer sequences are provided in Supplementary Table S1.

### Western blot

To begin with, cells were lysed using RIPA lysate (Epizyme, Shanghai, China) while being kept on ice. The protein concentration was detected using the BCA protein quantification kit (Epizyme, Shanghai, China). Next, gels were prepared and 20 μg of protein was loaded for electrophoresis using the PAGE Rapid Gel Preparation Kit (Epizyme, Shanghai, China). The protein gel was then transferred to a PVDF membrane. After the membrane transfer, the PVDF membrane was soaked in TBST solution containing 5% skimmed milk for 1 h at room temperature. The primary antibody was added and incubated overnight at 4 °C in the refrigerator. The next day, the membranes were incubated with HRP-labeled secondary antibody at room temperature for 1 h. Finally, the proteins on the membrane were visualized using an ECL ultrasensitive luminescent solution (Kemix, Zhengzhou, China).

### Cell counting kit-8 (CCK-8)

Cells were inoculated into 96-well plates at a density of 2 × 10^3^ cells/mL and incubated at 37 °C in a 5% CO_2_ incubator. After 24, 48, 72, and 96 h, 10 μl of CCK-8 (beyotime, Shanghai, China) reagent was added to each well. The plates were then incubated for 2 h, and protected from light, after which the absorbance at 450 nm was detected.

### Scratch assay

Cells were inoculated into 6-well plates at a density of 5 × 10^5^ cells/ml. When the cell growth reached 90%, three parallel lines were gently scratched on the bottom of the plate using a 200 μl sterile pipette tip. The healing process of the scratches was observed and photographed under a microscope at both 1 and 24 h.

### Transwell assay

For the invasion assay, Transwell chambers (Corning, New York, USA) lined with Matrigel basement membrane matrix (Solarbio, Beijing, China) were used. The cells were inoculated at a density of 2.5 × 10^5^ cells/ml in the upper chamber without serum. The upper chamber was then placed in the lower chamber containing 20% fetal bovine serum medium and incubated at 37 °C in a 5% CO_2_ incubator. After 24 h, the upper chamber was taken out, and the cells were fixed with 4% paraformaldehyde and then stained with 0.1% crystal violet solution. The results were observed under a microscope, and photographs were taken after drying the chambers. The migration assay followed the same procedure, except the Matrigel basement membrane matrix was not added.

### Immunofluorescence (IF)

Around 1 × 10^5^ cells were added to each well of 6-well plates that had cell crawlers (Biosharp, Hefei, China) lining the bottom. The plates were then placed in a 37 °C, 5% CO_2_ incubator for incubation. The following day, the cells were fixed with 4% paraformaldehyde for 15 min and then treated with 0.5% Triton X-100 (Solarbio, Beijing, China) for 15 min to make the membrane permeable. The cells were then blocked with an immunofluorescence special blocking solution for 1 h, after which a primary antibody was added and the cells were left to incubate overnight in a 4 °C refrigerator. Next, a FITC-labeled fluorescent secondary antibody was added and incubated in the dark for 1 h. The cells were then stained with a DAPI nuclear-staining reagent (Solarbio, Beijing, China) for 10 min. Finally, the cell crawlers were sealed with an anti-fluorescence quenching sealer and photographed under a fluorescence microscope as soon as possible.

### Animal experiments

Twelve 6-week-old female BALB/c nude mice were randomly divided into two groups. Each mouse was injected subcutaneously with either 3 × 10^6^ KYSE150 cells overexpressing IL-1β or control cells in the right hypochondriac region. The mice were observed for subcutaneous tumor formation one week after injection, and the tumor volume was measured and recorded twice a week. Tumor volume was calculated by the formula: 1/2 × *L* × *W*^2^ (*L*: long diameter, *W*: short diameter). After 28 days, the mice were euthanised, and the tumors were removed and photographed. Another twelve 6-week-old female BALB/c nude mice were randomly divided into two groups and injected with either 1 × 10^6^ KYSE150 cells overexpressing IL-1β or control cells into the tail vein. One month later, the mice were dissected for lung weighing and HE staining. The Zhengzhou University Animal Care and Use Committee approved all animal studies (ZZU-LAC20230728[08]).

### Statistical analysis

The data obtained from the experiment was analyzed using SPSS21.0 software and GraphPad Prism 7. All experiments were repeated at least three times and results are shown as mean ± standard deviation. The independent sample t-test was used for the statistical analysis of the data of two independent samples that conformed to the normal distribution, the rank sum test was used for the data of the two independent samples that did not conform to the normal distribution, the one-way ANOVA was used for the comparison of multiple quantitative data, and Fisher exact test was used for the statistical analysis of the clinicopathological parameters in categorical date. *P* < 0.05 was considered statistically significant.

### Supplementary information

The primer sequences used in this study

The supplementary figures used in this study

original western blots

## Data Availability

All data are available within the article and supplementary files, or from the authors upon reasonable request.
