# Supplementary material for: IL-1β promotes esophageal squamous cell carcinoma growth and metastasis through FOXO3A by activating the PI3K/AKT pathway
Source: Cell Death Discov. 2024 May 18;10:238. doi: 10.1038/s41420-024-02008-0 (PMC11102492; doi:10.1038/s41420-024-02008-0)
Supplement: Supplementary file 1 — The primer sequences used in this study [file 41420_2024_2008_MOESM1_ESM.docx]

Table S 1. The primer sequences used in this study.

| Primers | Forward | Reverse |
| --- | --- | --- |
| β-actin | GTGGCCGAGGACTTTGATTG | CCTGTAACAACGCATCTCATATT |
| IL-1β | GGACAGGATATGGAGCAACAAGTGG | TCATCTTTCAACACGCAGGACAGG |
| FOXO3A | TGGCAAGCACAGAGTTGGATGAAG | CATATCAGTCAGCCGTGGCAGT TC |
