# Supplementary material for: IL-1β promotes esophageal squamous cell carcinoma growth and metastasis through FOXO3A by activating the PI3K/AKT pathway
Source: Cell Death Discov. 2024 May 18;10:238. doi: 10.1038/s41420-024-02008-0 (PMC11102492; doi:10.1038/s41420-024-02008-0)
Supplement: Supplementary file 2 — The supplementary figures used in this study [file 41420_2024_2008_MOESM2_ESM.docx]

Supplementary Figure1.


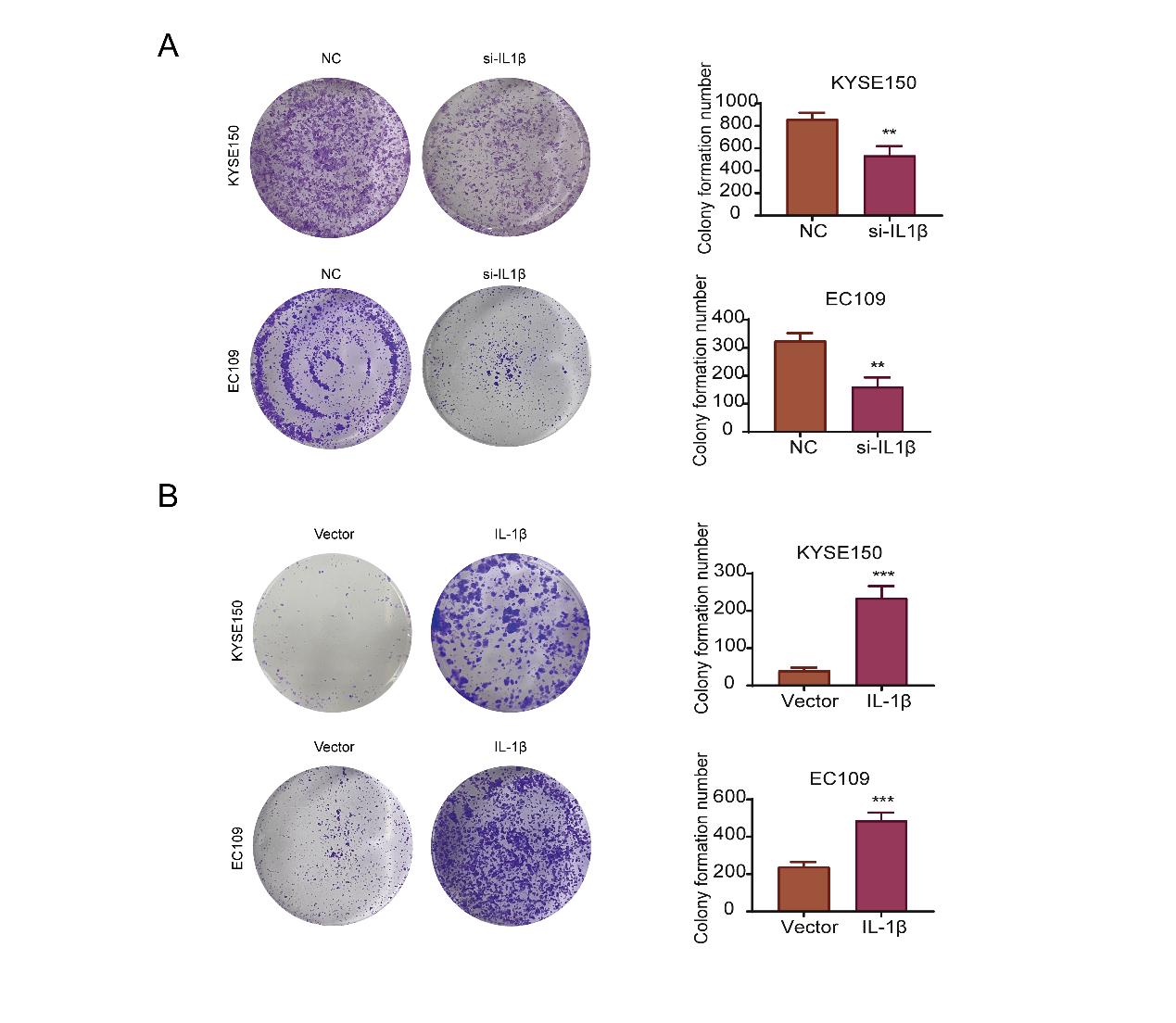


Supplementary figure 1. (A-B) The effects of knockdown and overexpression of IL-1β on cell clone formation were assessed by the clone formation assay.

Supplementary Figure2.


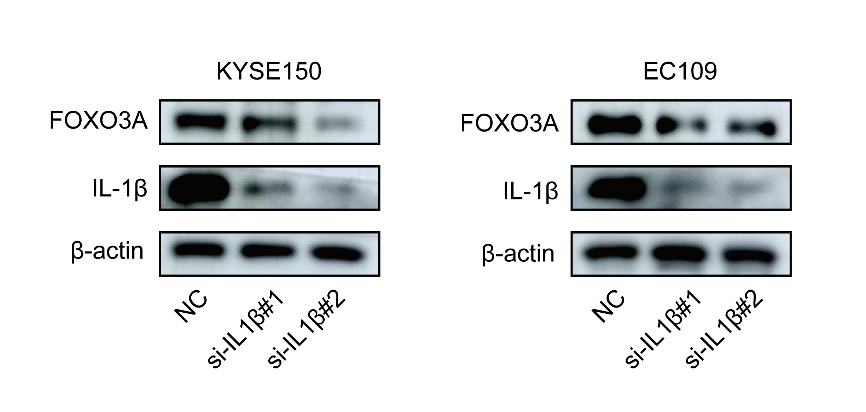


Supplementary figure 2. Western blot was used to detect the effect of IL-1β knockdown on the expression level of FOXO3A protein in KYSE150 and EC109.

Supplementary Figure3.


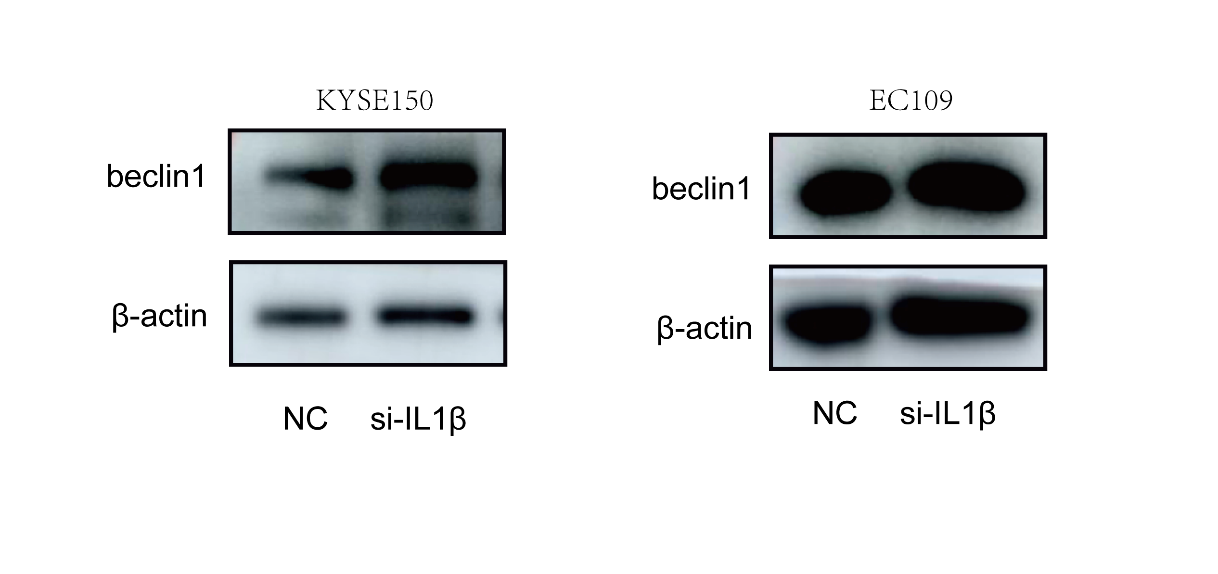


Supplementary figure 3. Western blot was used to detect the effect of IL-1β knockdown on autophagy-related protein beclin1 in KYSE and EC109.
