# Supplementary figures and images for: IL-1β promotes esophageal squamous cell carcinoma growth and metastasis through FOXO3A by activating the PI3K/AKT pathway
Source: Cell Death Discov. 2024 May 18;10:238. doi: 10.1038/s41420-024-02008-0 (PMC11102492; doi:10.1038/s41420-024-02008-0)

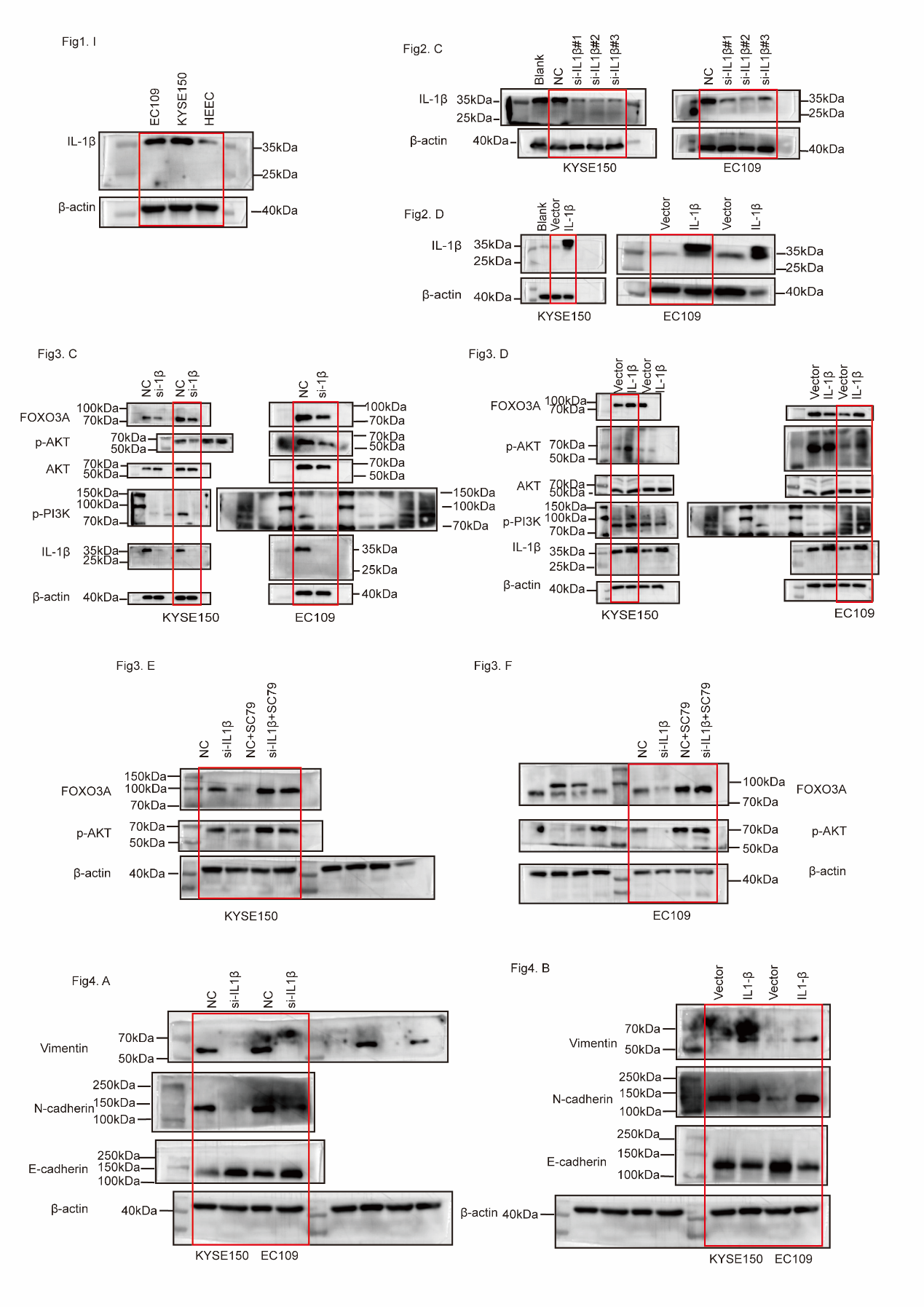


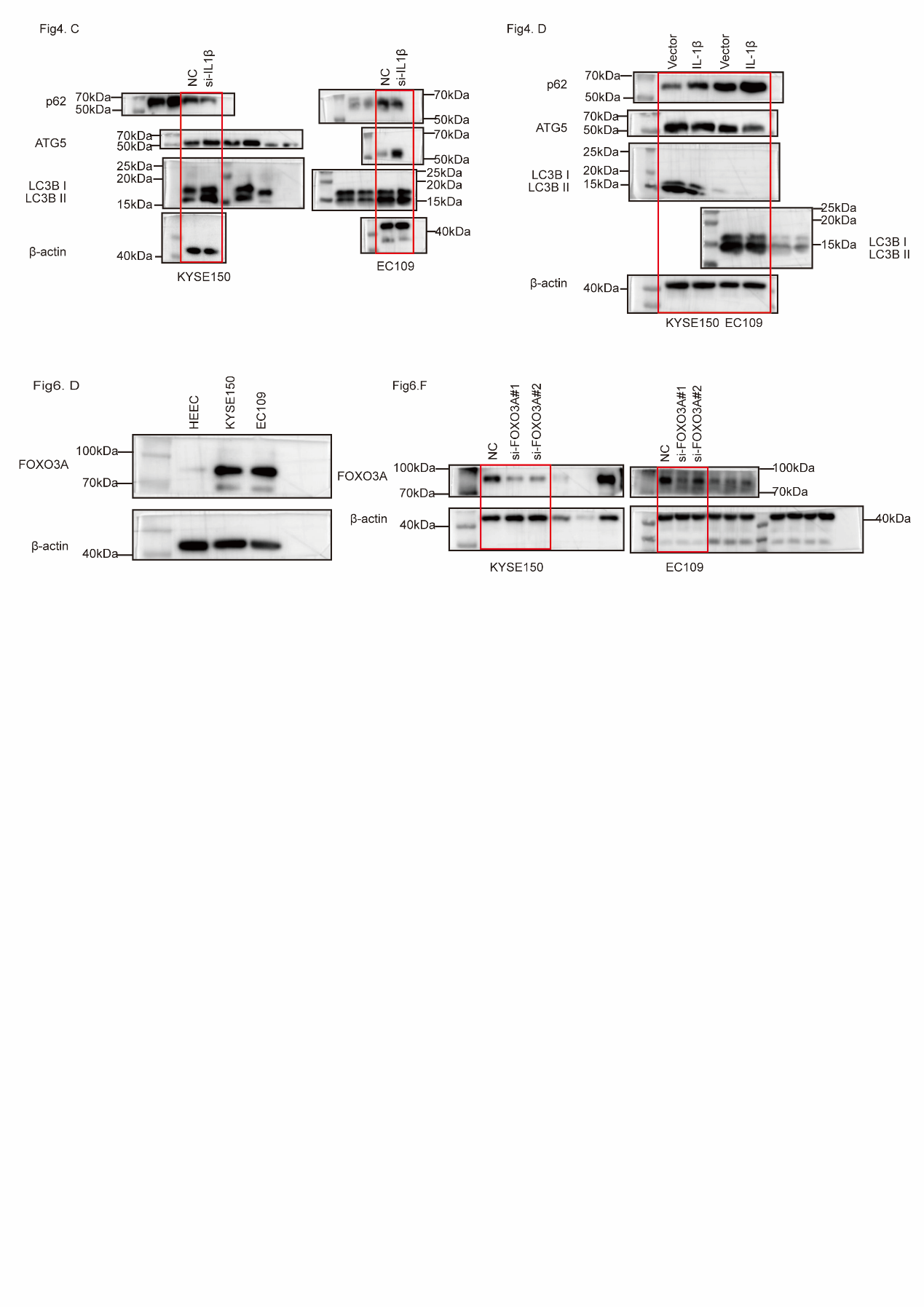

Supplement: Supplementary file 3 — original western blots [file 41420_2024_2008_MOESM3_ESM.docx]
